# Changes in Selected Biochemical Markers of Honey Bees Exposed to Fermented Common Tansy Solution (*Tanacetum vulgare* L.)

**DOI:** 10.3390/ani14192857

**Published:** 2024-10-04

**Authors:** Natalia Białecka, Klaudia Garbacz, Ewelina Berbeć, Agnieszka Murawska, Beata Madras-Majewska, Paweł Migdał

**Affiliations:** 1 Department of Bees Breeding, Institute of Animal Husbandry and Breeding, Wroclaw University of Environmental and Life Sciences, 38C Chelmonskiego St., 51-630 Wroclaw, Poland; 2 Apiculture Division, Institute of Animal Sciences, Warsaw University of Life Sciences, 166 Nowoursynowska St., 02-787 Warsaw, Poland

**Keywords:** biochemical markers, biopesticides, honey bee, *Tanacetum vulgare*

## Abstract

**Simple Summary:**

In agriculture, plant protection products are used to improve crop yields. They pose a threat to organisms that are not the target of their control. For this purpose, biopesticides have been used, which are supposed to show less toxicity to the environment and non-targeted organisms. On the other hand, natural pesticides can also show toxicity to insects. The honey bee is one of the insects that is still exposed to contact with residues of plant protection products, which poses a threat to this organism.

**Abstract:**

Honey bees use pollen and nectar from flowers to produce food. Because they often forage on crops, they are at risk of being exposed to plant protection products (PPPs), both directly and in stored food. Due to the adverse effects of synthetic PPPs on pollinators, biopesticides may be a viable alternative. Common tansy extract is used as one of the natural substitutes for synthetic pesticides. In our study, the effect of fermented common tansy extract on aspartate aminotransferase (AST), alanine aminotransferase (ALT), alkaline phosphatase (ALP), and gamma-glutamyl transpeptidase (GGTP) activity and the concentration of triglycerides (TGs), total protein (TP), total antioxidant status (TAS), and glucose in honey bee workers’ hemolymph was assessed. These biochemical markers give valuable information about the immunity, detoxification, and nutrition of a bee’s body. Caged bees were given tansy extract added at various concentrations in sugar syrup for 24 h. Then, they were provided with only sugar syrup. After 7 days of the experiment, hemolymph was collected and analyzed. We observed changes in the activity of AST, ALT, GGTP enzymes and TG, TP, and glucose levels, but not all changes were statistically significant. In terms of AST activity, statistically significant differences were found. All groups tested, including the negative control group, showed reduced enzyme activity values compared to the positive control group. In TG concentration, differences were observed between the groups receiving 2% extract and 1% ethanol. Glucose levels differed between the groups receiving 1% extract and 2% extract and between the positive control group and 1% extract. Bee body proper functioning is affected by changes in enzyme activity, especially those responsible for immunity and detoxification, such as AST, ALT, ALP, and GGTP. Despite the short time of bees’ exposure to the agent, the results of study show visible effects. Our results provide a basis for further research on the impact of tansy extract on honey bees.

## 1. Introduction

**Figure 1:**
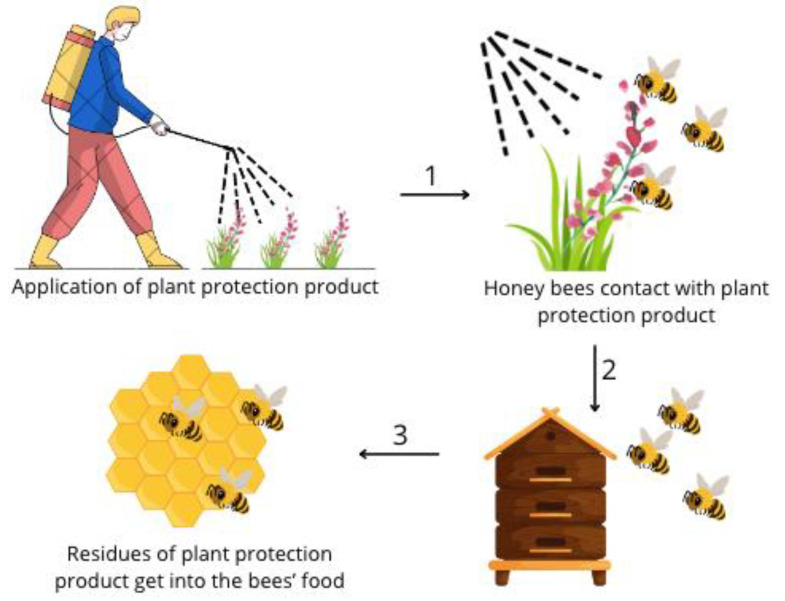
Diagram of the transport of plant protection product residues from flower pollen and nectar to bees’ food.

Plant protection products (PPPs) have significant importance and are currently widely used in agricultural production to reduce losses and improve crop quality. Unfortunately, PPPs have an adverse effect on the environment, partly because they can accumulate in ecosystems and affect non-target organisms [1,2,3] (Figure 1).

**Figure 2:**
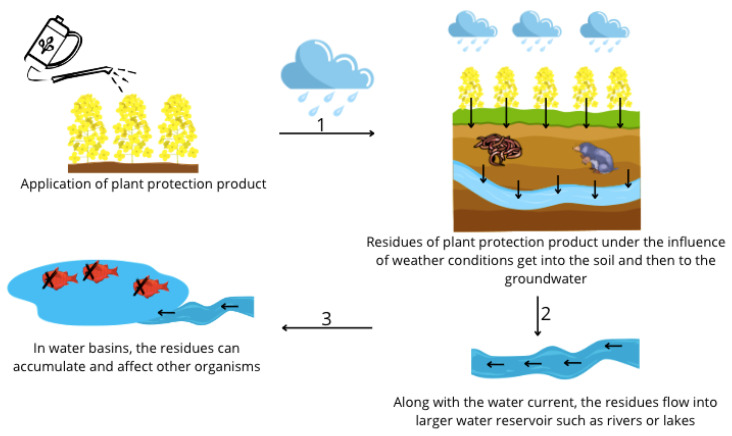
Diagram of the transport of plant protection product residues to the soil and water reservoirs.

The nectar and pollen utilized by honey bees to produce food and store in the hive contain residues of PPPs (Figure 2) [4]. Therefore, honey bee workers are likely to be constantly exposed to various PPPs. Depending on a compounds concentration and toxicity, it can be a threat to them [5,6]. To reduce the risk that occurs with the use of synthetic PPPs, natural pesticides (biopesticides) are gaining increasing interest. They are considered environmentally friendly, less toxic, and show lower levels of pest resistance. At the same time, they are generally assessed as safer for non-target organisms than synthetic pesticides [7] and have a short persistence in the environment [8]. Biopesticides contain a wide range of substances of natural origin, including botanical compounds such as secondary plant metabolites, essential oils, and plant extracts, and microorganisms such as fungi, bacteria, and viruses [9,10,11]. Essential oils and extracts are obtained from, for example, buds, flowers, seeds, and leaves, and they contain biologically active phytochemicals used in the pharmaceutical, cosmetic, food, and agricultural industries [12]. Plant extracts are a mixture of volatile and non-volatile substances with different properties. They often have a richer composition than essential oils and are a source of unsaturated fatty acids, oleoresins, polyphenols, flavonoids, phytosterols, terpenes, phenylpropane derivatives, antioxidants, active ingredients, and natural dyes or vitamins [13].

The biological activity of oils and extracts depends on their composition, primarily on the species of the used plant, their growth stage, and the place of collection [10,14,15]. Although essential oils and plant extracts are generally considered safer than their synthetic counterparts, their usage also carries risks. Due to wrong selection of the agent and improper preparation, they can exhibit significant toxicity [10].

One of the substances being studied as a natural substitute for synthetic pesticides is common tansy (*Tanacetum vulgare* L.) extract. Common tansy is a plant abundant in phenolic acids and flavonoids, which have medicinal and pharmacological properties [16]. It occurs naturally in Europe and Asia and has been introduced to North America [17]. The main components of common tansy essential oil are α-thujone, β-thujone, chrysanthemum acetate, and camphor [18]. Studies show that tansy extracts have antibacterial, anti-inflammatory, and antifungal properties [19]. Due to its strong smell, it is also used as a natural repellent [20,21] and insect-preventing [22] agent. Due to the use of plant extracts (including those derived from common tansy) to repel insects, concerns about their safety for honey bees have arisen.

Some essential oils affect the metabolic, biochemical, physiological, and behavioral functions of insects [23]. Simultaneously, they can have a significant effect on the larval forms of insects [24]. A study by Magierowicz (2020) found that common tansy essential oil and its components, such as β-thujone and camphor, can reduce the pupation of Acrobasis advenella larvae and increase their mortality [24]. However, there is a lack of information about the effect of this extract on the biochemical markers of the honey bee, which are a valuable element in the assessment of a bee’s health due to the fact that they play a significant role in immunity and detoxification [25]. Of great importance are also non-enzymatic markers, such as glucose, triglyceride, and protein levels, which, together with the enzymes, take part in defense against environmental threats [26].

This study aimed to assess how the fermented *T. vulgare* extract at different concentrations affects selected biochemical markers of honey bee workers.

## 2. Materials and Methods

### 2.1. Research Material

**Figure 3:**
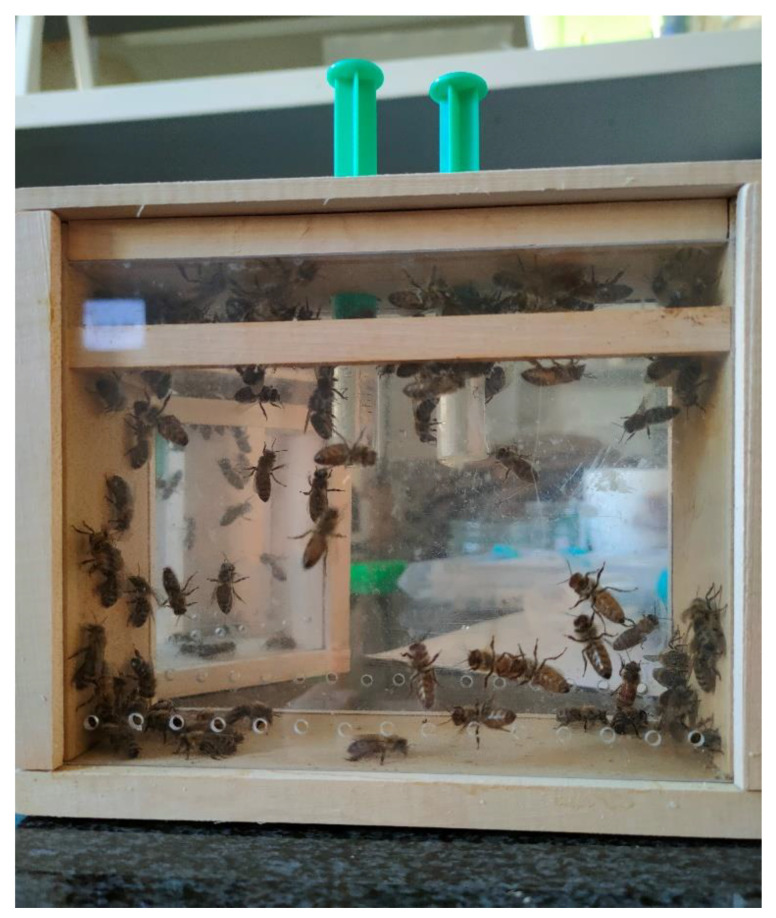
Experimental cage with food dispenser.

The research material was 1-day-old Carniolan honey bee workers (*Apis mellifera carnica*). On the 20th day of development, a brood previously reared naturally in a hive was placed in an incubator (34.4 °C ± 0.5 °C, relative humidity of 70% ± 5%, food ad libitum). Immediately after emerging, workers were randomly placed in wooden–plastic cages (20 × 15 × 7 cm) with two food dispensers (5 mL) (Figure 3). 

The extract used in the study (Tansy Extract, Target, Kartoszyno, Poland) was derived from the fermentation process of common tansy with the participation of *Lactobacillus* and *Bacillus* bacteria, according to the manufacturer’s description. The product is intended for use on ornamental, vegetable, fruit plants, and lawns to combat grubs, weevils, wireworms, and other pests.

### 2.2. Extract Exposure

The bees were divided into 7 groups, including 3 experimental groups provided with the fermented common tansy extract added the sucrose syrup in different concentrations (0.5, 1, or 2%) to and 4 control groups (3 negative control groups provided with sucrose syrup and ethanol in concentrations corresponding to tansy extract concentration, and 1 positive control group with sucrose syrup without additives). Ethanol was chosen as a negative control because it is the most commonly used carrier of active substances in plant extracts. This was to check whether the factor affecting the bee was not only the carrier of the substance. It is called a negative control group because the tested preparation is not administered. The molar concentration of sucrose syrup was 1 mol/dm^3^. Each group consisted of 10 cages, each with 100 workers. This gave 7000 bees for the entire experiment. Honey bee workers from the experimental groups were exposed to syrup with added tansy extract for 24 h. After 24 h, the bees received sugar syrup without additives ad libitum for 6 days until the end of the experiment. The extract concentrations corresponded to the dose recommended by the manufacturer and a dose 4 times lower than the recommended one. In this way, we assessed the scenario of when a bee has acute contact with the tested substance. The positive control group received 1 mol/dm^3^ sugar syrup for 7 days, while the negative control group received sugar syrup with the addition of ethanol at a concentration of 0.5, 1, or 2% for 24 h and then received only sugar syrup. 

### 2.3. Hemolymph Collection and Analysis

Hemolymph was collected using glass capillaries after 7 days of the experiment. For this purpose, the bee’s antennae were removed and the abdomen was pressed from the end towards the head to allow the hemolymph to flow out according to the method of Migdał et al. (2020). From randomly selected bees from each group, hemolymph was collected to obtain 5 capillaries for each group in 5 repetitions. The capillaries (5 pieces of 20 µL) were placed in an Eppendorf tube with 200 µL of milliQ water. During the collection, the samples were kept on cooling blocks to prevent melanization and then stored at −80 °C until analysis [27].

### 2.4. Analysis of Selected Biochemical Markers

The methodology from the publication by Migdał et al. (2023) was used to analyze the markers. All analyses were performed with a Pentra 400 biochemical analyzer (HORIBA ABX Diagnostics, Montpellier, France), device-compatible reagents, and the original manufacturer’s ready-made kits [28].

Total protein was determined using the Lowry method. In detail, 10 µL of hemolymph was added to 10 µL of copper reagent and incubated for 10 min at 25 °C. Then, 40 µL of Folin reagent (1:17) was added and incubated for 5 min at 55 °C. The obtained samples were analyzed spectrophotometrically (Eppendorf BioPhotometer, Sigma-Aldrich, Berlin, Germany) to measure the absorbance at 650 nm [29,30]. Glucose and triglyceride concentrations were analyzed colorimetrically using Cormay monotests (Lublin, Poland) according to the manufacturer’s procedure [31,32].

### 2.5. Statistical Analysis

All analyses were performed in Statistica (version 13.3). The Shapiro–Wilk test was performed to check the normality of the data distribution. The Kruskal–Wallis with Dunn’s test for multiple comparisons was used to assess differences between groups for data not derived from a normal distribution. For data derived from a normal distribution, analysis of variance with post hoc Fisher’s NIR was performed. The level of significance was *p* = 0.05.

## 3. Results

### 3.1. Results of Selected Biochemical Markers

**Figure 4:**
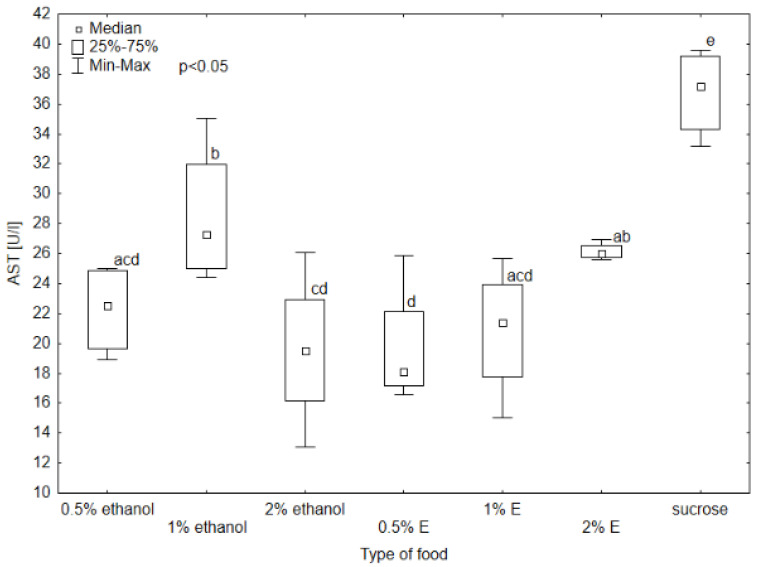
Aspartate aminotransferase (AST) activity in bee’s hemolymph—ethanol—indicates the percentage of ethanol added to food as a negative control, E—indicates the percentage of tansy extract (*Tanacetum vulgare* L.) added to sucrose syrup. Sucrose—a positive control receiving the base syrup at a concentration of 2 M. The whiskers at all box-plots indicate the variability in values and the minimum and maximum values. The lower frame of the box indicates the first quartile (Q1), and the upper frame the third quartile (Q3). Small letters at the boxes indicate the results of the Kruskal–Wallis test. Different letter symbols between groups indicate statistically significant differences.

**Figure 5:**
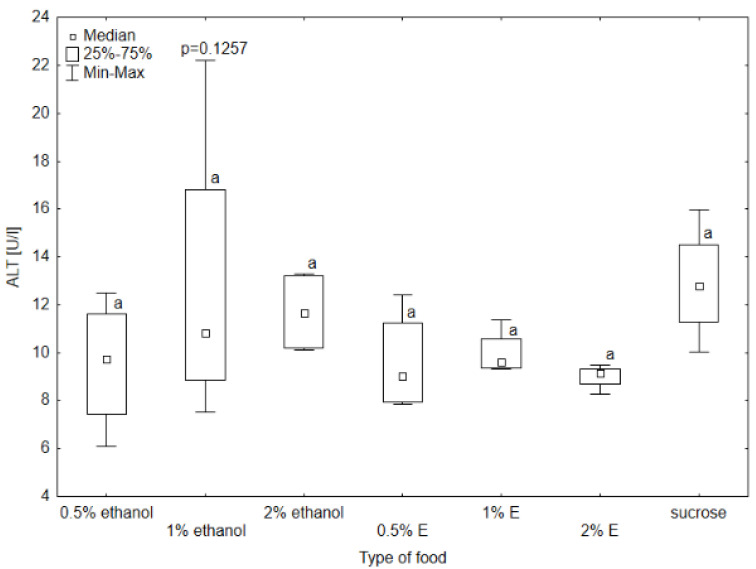
Alanine aminotransferase (ALT) activity in bee’s hemolymph—ethanol—indicates the percentage of ethanol added to food as a negative control, E—indicates the percentage of tansy extract (*Tanacetum vulgare* L.) added to sucrose syrup. Sucrose—a positive control receiving the base syrup at a concentration of 2 M. The whiskers at all box-plots indicate the variability in values and the minimum and maximum values. The lower frame of the box indicates the first quartile (Q1), and the upper frame the third quartile (Q3). Small letters at the boxes indicate the results of the Kruskal–Wallis test. Different letter symbols between groups indicate statistically significant differences.

**Figure 6:**
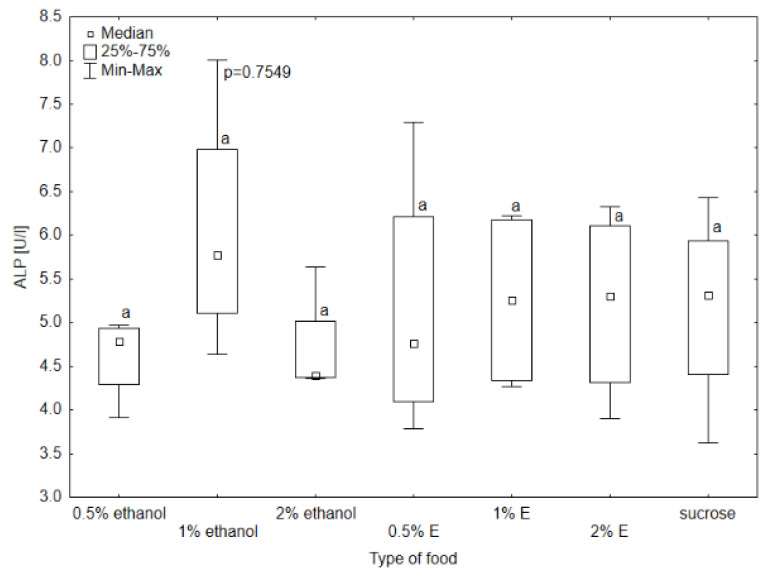
Alkaline phosphatase (ALP) activity in bee’s hemolymph—ethanol—indicates the percentage of ethanol added to food as a negative control, E—indicates the percentage of tansy extract (*Tanacetum vulgare* L.) added to sucrose syrup. Sucrose—a positive control receiving the base syrup at a concentration of 2 M. The whiskers at all box-plots indicate the variability in values and the minimum and maximum values. The lower frame of the box indicates the first quartile (Q1), and the upper frame the third quartile (Q3). Small letters at the boxes indicate the results of the Kruskal–Wallis test. Different letter symbols between groups indicate statistically significant differences.

**Figure 7:**
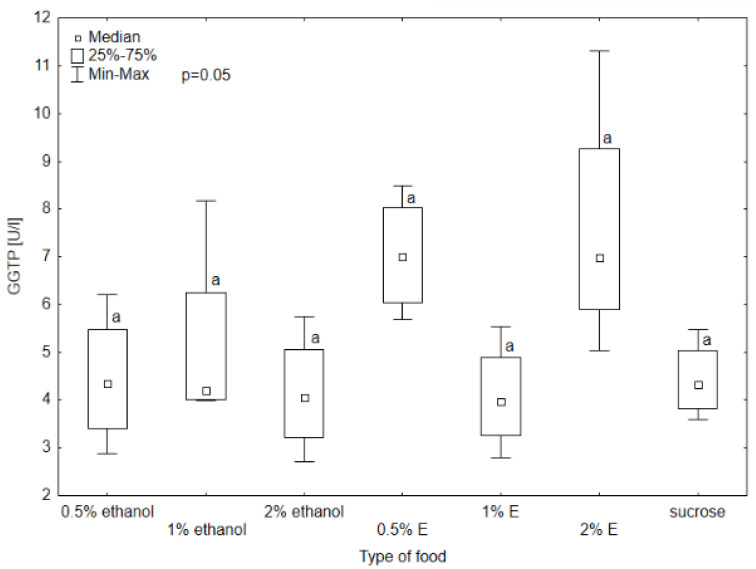
Gamma-glutamyl transpeptidase (GGTP) activity in bee’s hemolymph—ethanol—indicates the percentage of ethanol added to food as a negative control, E—indicates the percentage of tansy extract (*Tanacetum vulgare* L.) added to sucrose syrup. Sucrose—a positive control receiving the base syrup at a concentration of 2 M. The whiskers at all box-plots indicate the variability in values and the minimum and maximum values. The lower frame of the box indicates the first quartile (Q1), and the upper frame the third quartile (Q3). Small letters at the boxes indicate the results of the Kruskal–Wallis test. Different letter symbols between groups indicate statistically significant differences.

Aspartate aminotransferase (AST) activity in the hemolymph of bees provided with sucrose syrup without additives was significantly higher than in all other groups (*p* < 0.05). The results also differed significantly in the case of the group receiving 1% and 0.5% ethanol (respectively 27.27 U/L and 22.39 U/L), 1% ethanol and 0.5% tansy extract (17.97 U/L), and 1% ethanol and 1% tansy extract (21.38 U/L) (Figure 4). The median AST activity in the hemolymph of the bees receiving 0.5% and 1% tansy extract was similar, and for 2% ethanol was at a similar level. Higher values were achieved in groups consuming 0.5% and 1% ethanol, with the value of 1% ethanol being visibly higher and reaching a value close to 2% tansy extract. In the case of alanine aminotransferase (ALT), alkaline phosphatase (ALP), and gamma-glutamyl transpeptidase (GGTP) activity, the values did not differ significantly among groups, so no relationship was observed between these enzymes’ activity and the type of food received by bees (*p* > 0.05). The highest variability in ALT activity occurred in the group receiving syrup with 1% ethanol added, with the highest value of 20.2 U/L. The lowest value was observed in the group receiving 0.5% ethanol (6.02 U/L). There was also noticeable variability in GGTP activity, with the highest value reaching 11.4 U/L and the lowest 2.82 U/L (Figure 5, Figure 6 and Figure 7).

### 3.2. Selected Non-Enzymatic Biochemical Markers

**Figure 8:**
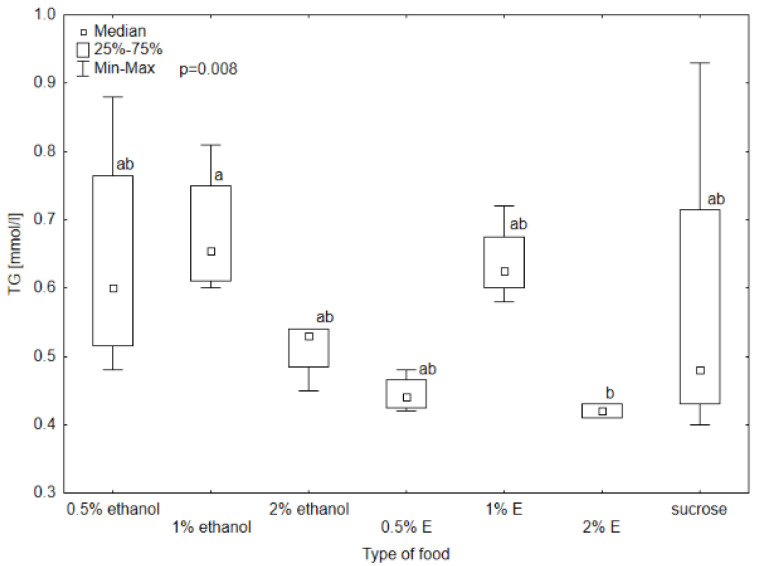
Triglyceride (TG) concentration in bee’s hemolymph—ethanol—indicates the percentage of ethanol added to food as a negative control, E—indicates the percentage of tansy extract (*Tanacetum vulgare* L.) added to sucrose syrup. Sucrose—a positive control receiving the base syrup at a concentration of 2 M. The whiskers at all box-plots indicate the variability in values and the minimum and maximum values. The lower frame of the box indicates the first quartile (Q1), and the upper frame the third quartile (Q3). Small letters at the boxes indicate the results of the Kruskal–Wallis test. Different letter symbols between groups indicate statistically significant differences.

**Figure 9:**
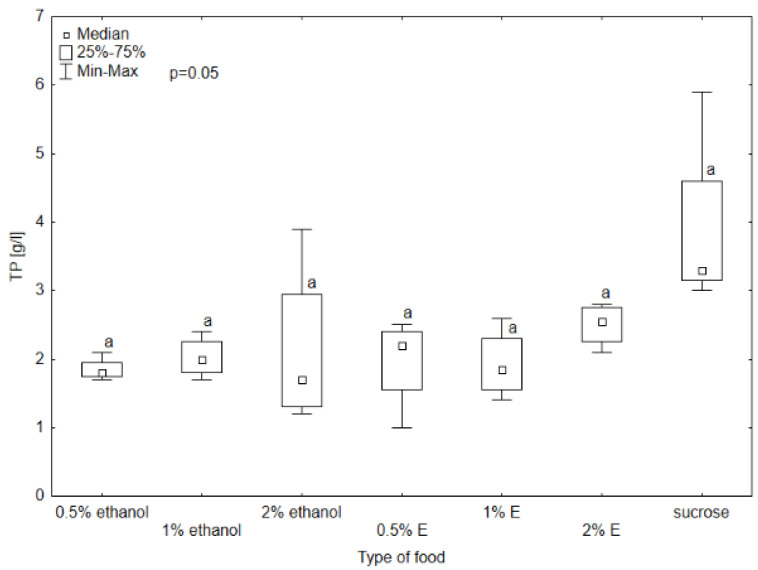
Total protein (TP) concentration in bee’s hemolymph—ethanol—indicates the percentage of ethanol added to food as a negative control, E—indicates the percentage of tansy extract (*Tanacetum vulgare* L.) added to sucrose syrup. Sucrose—a positive control receiving the base syrup at a concentration of 2 M. The whiskers at all box-plots indicate the variability in values and the minimum and maximum values. The lower frame of the box indicates the first quartile (Q1), and the upper frame the third quartile (Q3). Small letters at the boxes indicate the results of the Kruskal–Wallis test. Different letter symbols between groups indicate statistically significant differences.

**Figure 10:**
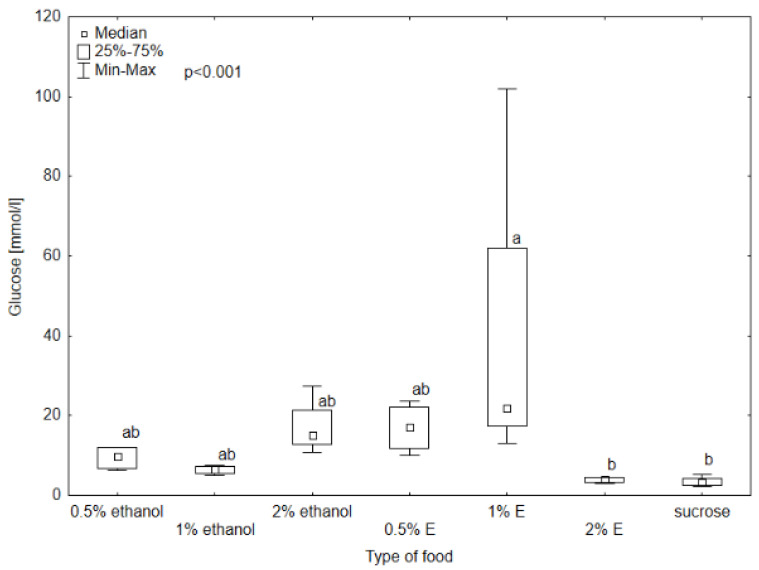
Glucose concentration in bee’s hemolymph—ethanol—indicates the percentage of ethanol added to food as a negative control, E—indicates the percentage of tansy extract (*Tanacetum vulgare* L.) added to sucrose syrup. Sucrose—a positive control receiving the base syrup at a concentration of 2 M. The whiskers at all box-plots indicate the variability in values and the minimum and maximum values. The lower frame of the box indicates the first quartile (Q1), and the upper frame the third quartile (Q3). Small letters at the boxes indicate the results of the Kruskal–Wallis test. Different letter symbols between groups indicate statistically significant differences.

**Figure 11:**
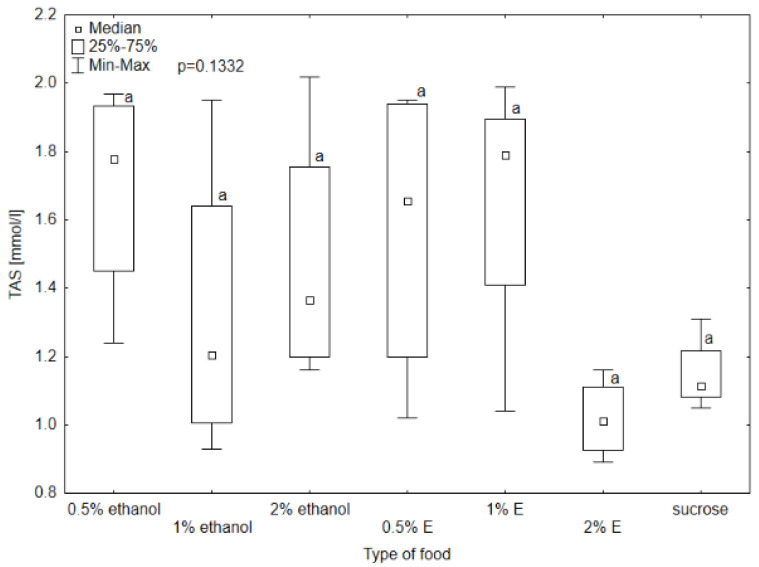
Total antioxidant status (TAS) concentration in bee’s hemolymph—ethanol—indicates the percentage of ethanol added to food as a negative control, E—indicates the percentage of tansy extract (*Tanacetum vulgare* L.) added to sucrose syrup. Sucrose—a positive control receiving the base syrup at a concentration of 2 M. The whiskers at all box-plots indicate the variability in values and the minimum and maximum values. The lower frame of the box indicates the first quartile (Q1), and the upper frame the third quartile (Q3). Small letters at the boxes indicate the results of the Kruskal–Wallis test. Different letter symbols between groups indicate statistically significant differences.

Triglyceride (TG) concentrations in the hemolymph of bees provided with 0.5, 1, and 2% tansy extracts were comparable to those receiving only sucrose solution, with the highest concentration for 1% tansy extract (0.63 mmol/L). The TG concentration was the lowest in the hemolymph of bees consuming 2% tansy extract. The Kruskal–Wallis test showed statistical significance between 2% tansy extract and 1% ethanol at the level of *p* = 0.008. The rest of the TG levels did not differ significantly. The glucose level reached the lowest value in the group receiving sucrose solution (3.52 mmol/L) and a very similar value to the group provided with 2% tansy extract (4.02 mmol/L). The value in the hemolymph of the bees consuming 1% tansy extract (21.60 mmol/L) reached the highest value of all groups and at the same time the highest variability occurred in this group. The levels in groups provided with 0.5 and 1% ethanol showed similar values to each other (respectively, 9.80 mmol/L and 6.53 mmol/L) but were visibly lower than the values of groups receiving 2% ethanol and 0.5% tansy extract (respectively, 15.31 mmol/L and 17.08 mmol/L). The Kruskal–Wallis test showed statistically significant differences between groups provided with sucrose solution and 2% tansy extract and 1% tansy extract, at the level of *p* < 0.001. In the case of total protein (TP) and total antioxidant status (TAS) concentrations no significant differences were observed between the type of food in all groups (*p* > 0.05). However, the TP concentration ranged from 5.9 g/L for the group receiving syrup without additives to 1 g/L for the group receiving 0.5% tansy extract (Figure 8, Figure 9, Figure 10 and Figure 11).

## 4. Discussion

There is little information about the impact of natural pesticides on honey bees. Some studies, however, show sublethal and fatal effects on bees after exposure to biopesticides [33]. These agents can affect not only pollinators’ mortality, but also some biochemical markers, which include enzymes, e.g., aspartate aminotransferase (AST), alanine aminotransferase (ALT), alkaline phosphatase (ALP), gamma-glutamyl transpeptidase (GGTP), and antioxidant enzymes. Studying these changes is important because metabolic detoxification is the main mechanism of the insect immune response. The participation of these enzymes in the detoxification of secondary plant metabolites and pesticides is why they are a frequently studied parameter in the honey bee [34,35].

In this study, we aimed to determine whether acute exposure to common tansy extract (a natural substitute for synthetic pesticide) affects honey bee workers. We decided to assess AST, ALT, ALP, and GGTP enzyme activity as well as total antioxidant status (TAS), total protein (TP), triglyceride (TG) concentration, and glucose levels in honey bee hemolymph. TAS, which stands for total antioxidant status, is a crucial indicator of the nutritional status of honey bees, similar to glucose, TP, and TG levels [32,36]. These markers were specifically chosen because the honey bee hemolymph contains significant enzymes originating from the fat body, which include aspartate aminotransferase, alanine aminotransferase, alkaline phosphatase, and gamma-glutamyl transpeptidase [37]. These enzymes are synthesized in the fat body and released into the hemolymph. The fat body tissue is the main storage site for nutrients and is responsible for innate immunity and detoxification [25,26,38]. The activity of the mentioned enzymes constitutes individual detoxification mechanisms responsible for metabolizing toxins from the organism [39]. That is why hemolymph is a key element in assessing the physiological state of honey bees.

In this study, AST activity in bee hemolymph was significantly lower in all groups compared to the group receiving sucrose solution without additives, while ALP, ALT, GGTP activity, and TAS remained at a similar level in all groups. The groups receiving syrup with added ethanol did not differ significantly from the other groups in terms of ALT, ALP, and GGTP activity, and had similar values. Significant differences occurred only in the case of AST activity in the group receiving syrup with added 1% ethanol and the other groups, without a positive control group. For non-enzymatic markers, all groups showed similar enzyme activity relative to each other. It is known that detoxification enzyme activity decreases in response to unfavorable environmental conditions, including parasitic activity and the impact of toxic substances, as well as physical stressors (for example, electromagnetic fields). In the study by Migdał et al., 2024, bees were exposed to an electromagnetic field of different intensity. TAS was lower compared to the control group in all groups except for the groups with the highest PE intensity and short exposure time [32]. The study by Bajda et al. (2014) showed reduced ALP, ALT and AST enzyme activity in response to the administration of amphotericin B (antifungal antibiotic) to bees at different concentrations. The longer the duration of antibiotic exposure, the less active the enzymes were compared to the groups receiving sugar syrup. The highest variability was shown in AST activity. The highest value reached 175 U/L in the control group, while in the study groups the values were close to 0 U/L for the antibiotic dose of 0.50 mg/mL and 10 U/L at the dose of 0.25 mg/mL [40]. In another study, bees exposed to imidacloprid at doses of 5 ppb and 200 ppb had reduced ALT, AST, and ALP activity regardless of age [41]. Increased ALP, AST, and ALT enzyme activities and glucose levels were obtained in the study by Skowronek et al. (2022) in response to the exposure of bees to CBD extract. The activity of all markers increased over experiment time compared to the control group, which may improve bee immunity [42]. In another study by Migdał et al. (2024), the effect of plant protection products, administered individually or in a mixture, on biochemical indicators and the mortality of honey bees was assessed. ALT activity was significantly higher compared to the control group in groups receiving pesticide mixtures at various dilutions. AST activity was also higher when the same mixtures were administered [43].

In this study, higher activity compared to the positive control group was observed in the TG concentration of the groups receiving 0.5%, 1%, and 2% ethanol and the group receiving 1% extract, while the glucose level was the lowest in the positive control group. All other groups showed higher glucose levels. Migdał et al.’s 2021 studies showed lower glucose levels in hemolymph in response to exposure to an electromagnetic field of different intensities. Instead, the TP concentration was the lowest in all control groups, and an increase in protein concentration was observed in the study groups with increasing exposure time. This may indicate increased immunological processes in response to external factors, affecting a bee’s body for longer. At the same time, proteins affect the activity of the antioxidant system, which also takes part in the body’s defense reactions. In the case of TG, the highest concentration was observed in the control groups. A decrease in triglyceride concentration was observed with longer exposures, but not all results were significant [44]. The results of the same team showed variability in glucose levels in studies from 2024. Glucose levels in the hemolymph after exposure to low-intensity PE for a short time initially increased, and after a longer exposure time decreased. However, at a higher PE intensity, glucose levels initially decreased, then increased, and decreased again. Glucose concentration increased at a higher dose of the agent [32].

Synchronously, bees use glucose to carry out their necessary metabolic processes. Similarly, regarding detoxification enzymes, it is produced in the insect’s fat body and is one of the main energy substrates during foraging and thermoregulation [45]. A reduced glucose level may indicate energy stress [42], which is why this level is a good indicator for analysis. Triglycerides are stored similarly and constitute the energy reserve of the bee organism [46]. They are used primarily during the winter period, when food availability is low. The amounts of these substances in the fat body decrease as the season progresses, and their amounts in the hemolymph increase [47]. Both of these indicators are related to a bee’s nutritional level, so their study helps assess the insect’s body condition. Another parameter is total protein. Studies show a constant positive correlation with the amount and quality of food consumed by a bee. A diet based on, for example, high-protein bee pollen increases the level of proteins in the hemolymph compared to a diet based on protein substitutes. In this study, a high level of proteins has a positive effect on the survival of bees during parasitic infections [48].

## 5. Summary and Conclusions

This study presents results on the acute exposure of honey bee workers to common tansy (*Tanacetum vulgare* L.) extract, which is used as a substitute for synthetic pesticides, and its effect on biochemical indicators. Due to changes in indicators characterizing the nutrition of the honey bee body, the effects may be long-term. AST showed reduced activity with 0.5%, 1%, and 2% tansy extracts, which means that the secretion of the enzyme could have been inhibited. Our findings indicate that acute exposure to tansy extract is not completely neutral for honey bees, even when exposed for such a short time (24 h). Further research is needed on the safety of biopesticides for honey bees, as well as their effects on bees. It is also important to investigate the long-term effects of the tested substances to better understand their impact. In order to properly represent natural conditions, further experiments should be carried out in semi-field and field conditions, including an entire bee colony.

## Data Availability

The datasets generated and/or analyzed during the current study are available from the corresponding author on reasonable request.
